# Leveraging Electronic Health Care Record Information to Measure Pressure Ulcer Risk in Veterans With Spinal Cord Injury: A Longitudinal Study Protocol

**DOI:** 10.2196/resprot.5948

**Published:** 2017-01-19

**Authors:** Stephen L Luther, Susan S Thomason, Sunil Sabharwal, Dezon K Finch, James McCart, Peter Toyinbo, Lina Bouayad, Michael E Matheny, Glenn T Gobbel, Gail Powell-Cope

**Affiliations:** ^1^ Center of Innovation on Disability and Rehabilitation Research Health Services Research and Development Department of Veterans Affairs Tampa, FL United States; ^2^ College of Public Health University of South Florida Tampa, FL United States; ^3^ Tampa VA Research and Education Foundation, Inc Tampa, FL United States; ^4^ VA Boston Healthcare System VA New England Healthcare System Department of Veterans Affairs West Roxbury, MA United States; ^5^ Muma College of Business University of South Florida Tampa, FL United States; ^6^ Geriatrics Research Education and Clinical Care Tennessee Valley Healthcare System Department of Veterans Affairs Nashville, TN United States; ^7^ Department of Biomedical Informatics Vanderbilt University Medical Center Nashville, TN United States; ^8^ Department of Biostatistics Vanderbilt University Medical Center Nashville, TN United States; ^9^ Division of General Internal Medicine Vanderbilt University Medical Center Nashville, TN United States; ^10^ Research and Development Service Tennessee Valley Healthcare System Department of Veterans Affairs Nashville, TN United States; ^11^ College of Nursing University of South Florida Tampa, FL United States

**Keywords:** natural language processing, pressure ulcer, risk assessment, spinal cord injury, text mining

## Abstract

**Background:**

Pressure ulcers (PrUs) are a frequent, serious, and costly complication for veterans with spinal cord injury (SCI). The health care team should periodically identify PrU risk, although there is no tool in the literature that has been found to be reliable, valid, and sensitive enough to assess risk in this vulnerable population.

**Objective:**

The immediate goal is to develop a risk assessment model that validly estimates the probability of developing a PrU. The long-term goal is to assist veterans with SCI and their providers in preventing PrUs through an automated system of risk assessment integrated into the veteran’s electronic health record (EHR).

**Methods:**

This 5-year longitudinal, retrospective, cohort study targets 12,344 veterans with SCI who were cared for in the Veterans Health Administration (VHA) in fiscal year (FY) 2009 and had no record of a PrU in the prior 12 months. Potential risk factors identified in the literature were reviewed by an expert panel that prioritized factors and determined if these were found in structured data or unstructured form in narrative clinical notes for FY 2009-2013. These data are from the VHA enterprise Corporate Data Warehouse that is derived from the EHR structured (ie, coded in database/table) or narrative (ie, text in clinical notes) data for FY 2009-2013.

**Results:**

This study is ongoing and final results are expected in 2017. Thus far, the expert panel reviewed the initial list of risk factors extracted from the literature; the panel recommended additions and omissions and provided insights about the format in which the documentation of the risk factors might exist in the EHR. This list was then iteratively refined through review and discussed with individual experts in the field. The cohort for the study was then identified, and all structured, unstructured, and semistructured data were extracted. Annotation schemas were developed, samples of documents were extracted, and annotations are ongoing. Operational definitions of structured data elements have been created and steps to create an analytic dataset are underway.

**Conclusions:**

To our knowledge, this is the largest cohort employed to identify PrU risk factors in the United States. It also represents the first time natural language processing and statistical text mining will be used to expand the number of variables available for analysis. A major strength of this quantitative study is that all VHA SCI centers were included in the analysis, reducing potential for selection bias and providing increased power for complex statistical analyses. This longitudinal study will eventually result in a risk prediction tool to assess PrU risk that is reliable and valid, and that is sensitive to this vulnerable population.

## Introduction

### Background

The pressure ulcer (PrU) is one of the most significant complications in veterans with spinal cord injury (SCI) in terms of morbidity, mortality, quality of life, and cost of care. The Veterans Health Administration (VHA) has the largest single network of SCI care in the United States providing a full range of care to more than 27,000 veterans. Services by the VHA are delivered through a “hub and spoke” system of care, extending from 24 regional SCI centers, offering primary and specialty care by interdisciplinary teams, to the 135 SCI patient-aligned care teams. These teams are typically comprised of an SCI coordinator (usually a social worker), a nurse, and physician, or support clinics at local VHA medical facilities [1].

The total cost of discharges for veterans with an SCI in the VHA who were discharged from a designated SCI system of care facility (ie, treating-bed section of SCI) with a primary admitting diagnosis of a PrU was over US $300 million for fiscal years (FYs) 2011-2012.  In this time period, veterans with SCI who were discharged from SCI facilities with the primary admitting diagnosis of a PrU—in the International Classification of Diseases, Ninth Revision, Clinical Modification (ICD-9-CM) 707.00-707.09 series—accounted for 64.1% (US $304M/US $474M) of the total costs for SCI discharges in the VHA (Veterans Health Administration, Managerial Cost Accounting System Discharge Pyramid Report, 2015.06.18). Stroupe and colleagues found that the cost of PrU-related hospitalizations for 150 veterans with SCI in the VHA over a 3-year period was approximately US $9 million and total health care costs were US $87,639 higher for patients with PrUs than those without (US $113,579 vs US $25,940, respectively) [2].

Current clinical practice guidelines for PrU prevention and treatment state that all patients should be assessed for risk of developing PrU using a valid, reliable, and sensitive tool [3,4]. The Braden Risk Assessment Scale [5,6], developed to predict risk in general inpatients, is presently used throughout the VHA for inpatients and outpatients. There is often a ceiling effect with most or all persons with SCI being identified at a high-risk level using the Braden Scale. Surveys conducted between 2008 and 2010 by the VHA External Peer Review Program at VHA Spinal Cord Injury and Disorder (SCI/D) Centers found that 91.3% of those measured in SCI were identified as being at high risk for PrU using the Braden Scale.

Reliability and sensitivity have not been fully established for existing tools in persons with SCI [7] despite the unique characteristics of this population (eg, lack of sensation and muscle wasting) [3]. Salzberg and colleagues devised a risk assessment scale specific to the SCI population; although used in a few SCI/D Centers [8], the psychometrics of this tool are limited [9,10].

This 5-year retrospective cohort study will leverage data available in the VHA’s electronic health record (EHR) to develop SCI-specific predictive models that can be used to better identify risk for PrUs. The new model will enable targeted prevention strategies, thereby reducing the burden of this serious complication on veterans, their caregivers, and the health care system.

### Scientific Rationale

Despite widespread implementation of risk assessment tools for PrUs, the incidence of PrUs among veterans with SCI has remained stable. Furthermore, there has been an increase in PrUs in the general population. Data from the Healthcare Cost and Utilization Project (HCUP), and Agency for Healthcare Research and Quality (AHRQ), identified a national increase of almost 80% in PrUs in 2006 compared with 1993; SCI was a frequent comorbidity (29.2%) of those hospitalized with a PrU [11].

In pilot work, we found that the majority of potential PrU risk factors identified in our review were available in the EHR in structured, unstructured, or semistructured data. Structured data, the easiest to extract, are precoded (ie, value or meaning is assigned) and stored in database tables such as the ICD-9-CM codes. Recent studies have shown that it is possible to develop valid risk models based on structured data in the EHR [12-14]. Semistructured data are text formatted into tables, templates, lists, and other documents. Extraction of such data is often difficult because it is commonly embedded within the unstructured clinical narrative and the presentation format is not standardized. Unstructured data are text that is formatted in traditional sentences and paragraphs. It is commonly considered the most difficult data to extract because natural language provides multiple methods of communicating the same essential information.

Recent studies have also shown that natural language processing (NLP) techniques can be used to extract risk and decision-making information from clinical text [15-17]. Natural language processing research focuses on developing computational models for understanding natural language [18]. The specific NLP technique employed varies depending on whether the text is semistructured or unstructured. The NLP techniques are used to extract and encode specifically targeted information in text (ie, information extraction [IE]) and convert it to structured data, which can then be readily used in risk assessment.

### Aims and Hypotheses

The goal of this study is to develop an improved SCI PrU risk model to better predict level of risk, guide preventive practices, and ultimately maximize the patient’s clinical outcomes. The resultant risk model, tailored to veterans with SCI, should promote adoption into clinical practice.

We will test two hypotheses to compare a model based on the commonly used Braden Scale versus (1) a risk model developed using structured data alone and (2) a model using both structured and NLP (ie, semistructured and unstructured) data. A third hypothesis will test the added value of NLP-based data over structured data. The hypotheses are as follows:

1. Hypothesis 1: The risk model using structured data alone will predict the development of PrUs better than the model based on the Braden Scale.

2. Hypothesis 2: The risk model based on combined structured and NLP data will predict the development of PrUs better than the model based on the Braden Scale.

3. Hypothesis 3: The risk model based on combined structured and NLP data will predict the development of PrUs better than the model based on structured data alone.

### Conceptual Framework

In developing this protocol, we employ the Aggregation, Organization, Reduction, Transformation, Interpretation, and Synthesis (AORTIS) model of understanding clinical summarization in both computer-independent and computer-supported clinical tasks [19]. Using this framework, “clinical summarization” is any act that can be carried out by a health care team member who utilizes patient and clinical information to create a structured data summary, which in turn supports clinical tasks.

**Figure 1:**
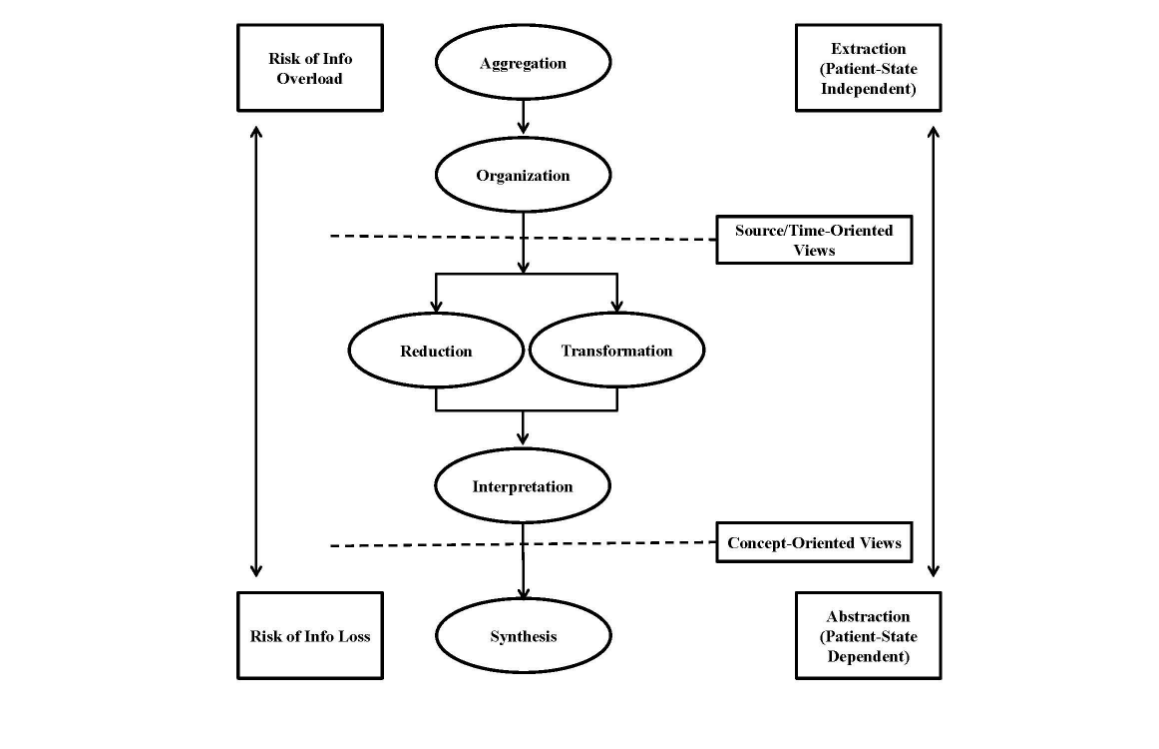
The model used to understand clinical summarization in both computer-independent and computer-supported clinical tasks. Info: information.

In our conceptual model, any or all of the steps to produce a concise and accurate summary could be performed by either a clinician or an automated system. The model was designed to be sequential, with the output from one step providing input into the next, and task dependent, with the content of each step varying based on the clinical task that the summary was designed to support (see Figure 1). In this study, we include many but not all of the steps of the model to employ AORTIS.

In AORTIS, data are organized around the specific clinical situation, development of a PrU. This expedites IE and clinical decision making but requires the application of a significant clinical knowledge base. The process of identifying and extracting the necessary data from structured data or text represents activities related to aggregation and organization. The application of NLP programs represents the reduction and transformation of complex text data, which was not readily computable, into structured data elements that could be included in prediction models. The development of prediction models represents the interpretation and synthesis of these data, which could be presented to clinicians for interpretation.

## Methods

### Overview

This is a 5-year longitudinal, retrospective, inception, cohort study targeting veterans with SCI. This research was approved by the Department of Research and Development, James A Haley Veterans’ Hospital, and the VHA Central Institutional Review Board. As described below, the study is ongoing. Each section describes the current status of the study activities as complete, ongoing, or planned.

### Expert Panel—Complete

An expert panel meeting was held in March 2014 to discuss PrU risk factors. The goal of the panel was to identify factors that were likely present in the EHR and could be used to develop improved risk models for PrU development. Participants included two physicians specializing in care for veterans with SCI, three nurse researchers, a wound care specialist, an SCI outcomes registry coordinator, a physical therapist, a dietitian, and a social worker. All members of the expert panel had multiple years of experience in managing persons with PrUs and SCI. Members of the study team also attended the expert panel meeting. Results of the expert panel were iteratively reviewed and refined by two additional physicians and a nurse specializing in research and treatment of PrUs in veterans with SCI.

### Variables Selection and Definition—Ongoing

The dependent variable for this study will be documentation of the occurrence of a PrU in the EHR within 1 year (ie, 1-year incidence risk of PrU). The definition of the presence of a PrU used for case identification is based on ICD-9-CM codes (707.0-707.9) and the application of the classification algorithm based on text analysis or a combination of these two sources, depending on what is found to be more accurate. The potential independent variables for this study include the risk factors that were identified through a literature review and validated by expert panel discussion. The final list of potential risk factors, with input from the expert panel, will be extracted from multiple tables in the EHR, or from semistructured and unstructured text notes, for use in analysis. Three sets of candidate predictors based on data sources will be generated to develop each risk model: (1) structured data extracted directly from the EHR, (2) Braden scores, and (3) a combination of existing structured data and data extracted from text through the NLP processes.

The investigators conducted a comprehensive literature review generating a list of approximately 50 potential independent variables or risk factors (eg, demographics, diseases status, comorbidities, health behaviors, psychosocial factors, and home care). This list was reviewed by the expert panel for logical consistency, completeness, clinical relevance, and whether they were modifiable or not. The panel identified high-priority risk factors and discussed practical considerations including where the risk factor might be located in the EHR, how reliable these data might be, and alternative ways to document risk factors. Subsequently, persistent moisture on the skin, the type of living situation of the patient, evidence of malnutrition, and documentation that the patient took steps to redistribute their weight during the day were targeted as risk factors available in unstructured data. Structured data contained elements of the Braden Scale, lab values, and other variables (see Table 1). Several coding systems are used to classify structured data variables, including the ICD-9-CM, Logical Identifiers Names and Codes (LOINC), and the Healthcare Common Procedural Coding System (HCPCS). International Classification of Diseases, Tenth Revision, Clinical Modification (ICD-10-CM) codes were not implemented in the VHA until after the end of this study and are not used.

### Sample and Sampling—Complete

Using VHA data in its Corporate Data Warehouse obtained through its Veterans Affairs Informatics and Computing Infrastructure (VINCI), we identified an inception cohort (N=12,344) of veterans with SCI who received care in the VHA in FY 2009, but had no PrU in the 12 months prior to 2009 based on recorded ICD-9-CM codes. This group was considered free of PrUs for the purpose of the study. All structured hospital discharges (N=4090), outpatient encounters (N=5,202,804), and text data (N=9,888,245) for the cohort for FY 2009-2013 (October 1, 2008-September 30, 2013) were obtained through the VINCI. Veterans from this cohort were classified as having a PrU (ie, PrU+) or not having a PrU (ie, PrU-) during the study period based on both structured and text data. A total of 3860 PrUs were identified based on structured data and 829 based on text data alone, resulting in a total incidence of 4689 (4689/12,344, 37.99%) across the study period.

**Table 1:** Risk factors categorized by structured, semistructured, and unstructured data sources.

Data source	Risk factor	Description or definition
Structured	Age	Age in years
	Gender	Male/female
	Race/ethnicity	Race/ethnicity
	Education level	Highest level of education
	VHA^a^ eligibility	VHA category, service connection
	Body weight	Body mass index, most recent weight, or change in weight
	Patient status	Inpatient/outpatient when pressure ulcer develops
	Pattern of preventive care	Consistency of annual well-patient exam
	Cognitive dysfunction	ICD-9-CM^b^ codes (ie, traumatic brain injury, dementia, and Alzheimer’s disease)
	Comorbidities—psychological	ICD-9-CM codes (ie, depression, anxiety, and posttraumatic stress disorder)
	Comorbidities—physical	ICD-9-CM codes (ie, diabetes mellitus, pulmonary disease, cardiac disease, renal disease, peripheral vascular disease, anemia, deep vein thrombosis, cancer, infection, lower extremity fractures, spasticity, autonomic dysreflexia, hypotension, and heterotopic ossification)
	Laboratory analysis	LOINC^c^ codes for lab values (ie, C-reactive protein, white blood cells, erythrocyte sedimentation rate, renal function, hemoglobin, hematocrit, albumin, and prealbumin)
	Medications	VHA formulary to identify chemotherapeutics, steroids, and benzodiazepines
	Surgery	ICD-9-CM codes of surgical procedures within previous 6 months of pressure ulcer development (administrative data)
	VHA-issued equipment	HCPCS^d^ codes for wheelchair cushion, bed support surface, and other equipment
	Telehealth	Distant observation of skin integrity
Semistructured	Motor/sensory assessment	American Spinal Cord Injury Association (ASIA) classification of SCI^e^ (eg, ASIA A= *Complete* injury of spinal cord)
	Level of injury	Cervical (C1-C8), thoracic (T1-T12), lumbar (L1-L5), and sacral (S1-S5)
	Alcohol use	Alcohol Use Disorders Identification Test—Consumption (AUDIT-C)
	Tobacco use	Current/former/nonsmoker
	Caregiver resources	Receiving aid and attendant, VHA-supported/reimbursed bowel/bladder care
	Duration of SCI	Years since injury/onset
	Sensation^f^	Ability to feel and relieve discomfort
	Moisture^f^	Exposure to moisture on trunk (ie, bowel/bladder leakage/accidents due to management or lack of containment, diarrhea, and diaphoresis)
	Mobility^f^	Ability to relieve pressure on high-risk areas (eg, trunk and heels)
	Activity^f^	Level of activity (ie, ability to get out of bed and ambulation)
	Nutrition^f^	Food intake malnutrition, undernutrition, and moderate/severe compromise
	Friction and shear^f^	Ability to minimize resistance between two parallel surfaces (ie, bed linens and skin)
	Living situation	Living arrangements (eg, alone, immediate family, extended family, roommate, and group setting)
Unstructured	Noncompliance with pressure redistribution	Patient refusal/decline to turn in bed
	Pain	Numeric pain rating scale (1-10) when pressure ulcer develops
	Pressure ulcer history	Previous pressure ulcer

^a^VHA: Veterans Health Administration.

^b^ICD-9-CM: International Classification of Diseases, Ninth Revision, Clinical Modification.

^c^LOINC: Logical Identifiers Names and Codes.

^d^HCPCS: Healthcare Common Procedural Coding System.

^e^SCI: spinal cord injury.

^f^Element of the Braden Scale.

### Data Extraction and Synthesis (Structured Data)—Ongoing

To determine whether the targeted structured variables were present in a patient’s record, we identified lists of codes (ie, ICD-9-CM codes) to operationally define the variables. When available, definitions employed by the VHA Support Service Center (VSSC), a centralized reporting portal, were used. The VSSC provides data summaries to VHA administrators and policy makers regarding business operations, clinical care, quality performance, and resource management. Otherwise, operational definitions were obtained from the AHRQ’s HCUP Clinical Classifications Software [20] or the Centers for Medicare and Medicaid Services definitions. Extraction routines were used to identify each occurrence of the variable in the patient’s record. These data were then transformed into single elements in the analytic data table based on clinically relevant temporal considerations. For example, some comorbidities such as diabetes were considered chronic conditions, while others such as fractures were framed in a 1-year period.

### Data Extraction and Synthesis (Unstructured and Semistructured Data)

We will employ NLP techniques to extract targeted information from the documents in the EHR. This will be accomplished using the following steps.

#### Step 1: Create Reference Standard—Complete

Large samples of documents (approximately 2000) will be extracted from the larger corpus of documents. A human-annotated (ie, chart review) corpus will be developed, which will be used as a reference standard to train and evaluate NLP algorithms. An annotation schema was developed, which defines the classes of concepts based on the variables reviewed by the expert panel. The concepts will be annotated in each document in the corpus by two clinicians and adjudicated by a third clinical expert. Interrater reliability will be evaluated by calculating interannotator agreement. Agreement between the two annotators will be evaluated by calculating the *F* measure, proposed by Hripcsak and Rothschild [21], against the final reference that will be adjudicated by our clinical expert.

#### Step 2: Extract Information From Unstructured and Semistructured Text Data—Ongoing

We will use the annotated reference set of documents to develop and evaluate distinct methods to extract information from unstructured and semistructured text.

##### Step 2a

The General Architecture for Text Engineering (GATE) software will be used to create an NLP pipeline to extract the information from the unstructured text data [22]. The results of the NLP pipeline will be iteratively compared to the annotated terms from the reference standard on 70% of the documents. The remaining 30% of the documents will be used to validate the best model. This will enable the pipeline to be iteratively refined to more accurately identify the potential predicators located in the narrative documents.

##### Step 2b

We will develop an NLP system for semistructured text data. The composition of semistructured text presents unique challenges to NLP, making information extraction especially challenging [23,24]. Semistructured text consists of short, simple phrases that are not grammatically correct.

A traditional grammatical NLP system may fail to negate the presence of moisture for the patient. While this “structure” aids readability, it hinders traditional grammatical NLP systems. However, this “structure” makes rule-based systems using regular expressions a viable alternative for extracting targeted information. Thus, we will employ a custom-developed, rule-based system using named entities- and structural-based regular expressions, which will also be developed and evaluated using an annotated reference set of documents.

### Data Analysis—Planned

Three separate predictive models of PrU occurrence based on each set of predictors in each hypothesis will be developed using R statistical program version 3.3.2 (The R Foundation). To prepare, the distribution of each continuous predictor will be examined and variable transformation will be carried out as appropriate to approximate normality (eg, log transformation of skewed data). However, to avoid information loss, no continuous variable will be categorized in an initial prognostic model. Using the generalized additive model (GAM) approach, a bivariate logistic regression will be performed to test potential nonlinearity between each continuous candidate predictor and the binary PrU outcome [25]. This will provide further guidance for appropriate transformation needed for the predictor.

The analytic data sample (N=12,344) will be randomly divided into two groups in the ratio of 70% (n=8641) for derivation and 30% (n=3703) for validation. The randomization will be stratified by site, age group, and gender to increase similarity in the distribution of both validation and derivation random samples.

### Model Development

An automated model selection procedure will be performed with the R package *glmulti* [26] to select variables and interaction terms in a logistic regression model of PrU status in the training sample. The automated model selection procedure will return a set of *n* best models, ranked by their information criteria values as well as estimating importance scores for the predictors. Thus, it offers us the flexibility to select from the set a more parsimonious or more intuitively reasonable final model that is context based. Another consideration is for the final prognostic model to include variables that could be easily assessed in practice and with minimal burden on the respondent SCI patients. The fully specified prognostic model will eventually be part of an automated decision support system.

The clinical utility of the risk scores will be evaluated for different risk thresholds and the sensitivity, specificity, positive predictive value, and negative predictive value will be included as performance measures in the automated output. In addition, a separate listing of risk factors will be provided for each risk model and ranked by their rounded Z-scores (or standardized regression coefficients). A higher Z-score implies a more “important” contribution of that variable in the model. These lists will assist the clinicians in prioritizing what variables to assess. Each product will be accompanied with appropriate complete operational definitions and scoring instructions.

Data analysis will be focused on testing two hypotheses that compare the ability of the current standard risk assessment measure—Braden Scale—with models based on structure data alone and in combination with data extracted from text. Comparisons will be made between a pair of risk models to test each hypothesis using exactly the same veterans from the validating sample. Our priority will be to develop models with low false positive rate (ie, the rate at which negative instances are incorrectly classified as having PrU). This consideration will guide how any two competing risk models (ie, classifiers) will be compared using the receiver operating characteristic (ROC) curve analysis. The logistic model will output proper probability scores from which a full ROC curve will be generated for each classifier with overlay in one plot. Generally, a higher calculated total area under the curve (AUC) implies a better prognostic performance. However, when comparing two classifiers, it is possible for the classifier with a higher total AUC to show poorer local performance in our priority region. Therefore, we will assess classifier performance in the overall AUC as well as partial AUC (pAUC) in our priority local region of low false positive rate. The classifier that has a higher sensitivity (ie, true positive rate) in the priority region has superior local performance. That is, an appropriate pAUC will also be computed in addition to the standard full AUC.

Both discrimination and calibration will be investigated as validation measures for each prognostic model using the validation sample. The better discriminating models have higher AUC and pAUC values based on their CIs (ie, better able to distinguish between patients with SCI who have and do not have PrU). To assess calibration, the predictions of each model will be compared with the veterans’ actual outcomes in the validation sample by performing the Hosmer-Lemeshow test using the hoslem.test function in the Resource Selection library of R. To ensure that the overall type I error rate of α is maintained, first, we will repeatedly sample from each model and calculate the Hosmer-Lemeshow *P* value 1000 times, and calculate the proportion of *P* values less than .05 (ie, type I error rate should be no greater than 5%).

A graphical approach will be used to compliment the Hosmer-Lemeshow test since it has been known to be sensitive to sample size. For the graphical method, first, the sample will be divided into 10 risk groups of equal size with each group having similar model predicted probabilities. Next, the observed proportions of PrUs will be plotted against predicted probabilities for these groups with one smooth curve fit (eg, lowess) as well as a linear fit to the data points. We anticipate that the observed and predicted occurrence of PrUs will be similar across the risk groups. A perfect calibration (ie, perfect agreement between the predicted and observed outcomes) will produce a calibration line with an intercept and slope of 0 and 1, respectively [27-29]. The smooth curve will be added to reveal differential calibration among risk deciles, if present.

We expect the validation study to span across different domains (eg, inpatient vs outpatient, site, age group, and gender) as this provided the strongest evidence of generalizability of the prediction rule to new patients [30]. Since stratified randomization ensured a similar case mix within each of our samples—derivation and validation—and both samples were representative of our population of interest, we believe that the results of our validation tests will serve as strong evidence of generalizability.

## Results

The study is ongoing with results expected in 2017. The expert panel met and reviewed the initial list of risk factors based on the literature review (see Table 1). They made recommendations for additions and deletions and provided insight into where, and in what format, the documentation of the risk factors might exist in the EHR. This list was then iteratively refined through review and discussion with individual experts in the field. The cohort for the study has been identified and all structured, unstructured, and semistructured data have been exported into a relational database for analysis. A description of the cohort is provided in Table 2.

The cohort is almost exclusively male (11,796/12,344, 95.56%), with 68.96% (8513/12,344) being white and 19.64% (2424/12,344) being black or African American. The mean age of the cohort was 58 years (SD 14) with more than half (6782/12,344, 54.94%) being between 50 and 69 years of age. Approximately half (6873/12,344, 55.68%) reported being married.

Annotation schemas have been developed, samples of documents have been extracted, and annotation and adjudication are ongoing. Table 3 outlines the variable targeted through the annotation process.

**Table 2:** Description of study cohort (N=12,344).

Characteristic		n (%)
**Gender**		
	Female	548 (4.44)
	Male	11,796 (95.56)
**Race**		
	Asian	74 (0.60)
	Black or African American	2424 (19.64)
	Native Hawaiian or other Pacific Islander	1 (0.01)
	White	8513 (68.96)
	Other	28 (0.23)
	Unknown by patient/missing	1214 (9.83)
**Ethnicity**		
	Hispanic or Latino	939 (7.61)
	Non-Hispanic or non-Latino	10,723 (86.87)
	Unknown by patient/missing	82 (0.66)
**Age group, years**		
	<21	24 (0.19)
	21-30	491 (3.98)
	31-40	824 (6.68)
	41-50	1854 (15.02)
	51-60	3753 (30.40)
	61-70	3120 (25.28)
	71-80	1540 (12.48)
	81-90	701 (5.68)
	>90	36 (0.29)
	Missing	1 (0.01)
**Marital status**		
	Single/never married	2354 (19.07)
	Married	6125 (49.62)
	Divorced	3097 (25.09)
	Widow/widowed	560 (4.54)
	Unknown/missing	187 (1.51)

**Table 3:** Pressure ulcer (PrU) annotation schemas.

Task	Variable	Attributes	Description	Example text spans
Annotation task 1	Pressure ulcer	Pressure ulcer	Text indicating presence of a PrU	Pressure ulcer, skin sore, decubitus ulcer
		Stage	Stage of the PrU	Stage 1, stage 2, unable to stage, stage 3-4
		Laterality	Right, left, bilateral, midline, not applicable, unspecified	Right, left, lower extremity
		Location	Anatomic location of PrU	Coccyx, heel, trochanter, sacrum, ankle, ischial
		Orientation	Medial, lateral, proximal, distal, dorsum, plantar, anterior, superior, posterior, inferior, unspecified	Bilateral
		Temporality	Date: date of examination Duration: span of time since PrU discovered	Sept 10, 2009, approximately one year
		Assertion	Modifiers: historical, recurrent, negated, hypothetical, not PrU, unspecified	Healed pressure ulcer, history of pressure ulcers, no pressure ulcers, if pressure ulcers develop
	Noncompliance	None	Documentation of noncompliance with pressure release	Patient refuses to turn because of pain
	In label	None	PrU mentions within section labels in documents (ie, are not indications of a PrU)	Pressure ulcer protocol Pressure ulcer education
Annotation task 2	Living situation (LS)	LS cue	Key phrase indicating presence of LS	Living at, living with, address, living arrangements
		LS main	Actual value assigned to LS (ie, alone, nuclear family, extended family, roommate, group, homeless)	Mother’s residence, girlfriend, half way house, caregiver, spouse
	Malnutrition (MN)	MN cue	Key phrase indicating presence of MN	Nutrition status:
		MN main	Actual value assigned to MN	Moderately compromised, severely compromised
	Moisture (MO)	MO cue	Key phrase indicating statement about MO	Moisture: Constantly moist:
		MO main	Text indicating presence of MO (ie, fecal, urinary, sweat)	Multiple loose stools, copious amounts foul purulent drainage, night sweats, perspiration, urine
		Assertion	Modifiers: asserted, historical, hypothetical, negation, not patient, uncertain	Was, prior medical history, nutrition risk, manage, []^a^, history of
Annotation task 3	Template components	Header	Title line naming the template	Braden Scale, ASIA^b^ score
		Item label	Label for individual assessment items	Moisture, turgor, color, temp
		Item score	Value (text or number) assigned to item	Moist, red, 4, 0
		Total label	Text identifying the summed score	Total score:
		Total score	Final value for the assessment	30, high risk
		Whole template	Offsets from block of text identifying template boundaries	132-165

^a^These empty brackets would be generated by the system and placed in a progress note when a check box was left blank in a template in the electronic medical record system.

^b^ASIA: American Spinal Cord Injury Association.

The annotation process was split into three tasks to allow for sampling of different types of documents, depending on the targeted variables. This also reduces the cognitive burden for the annotators in each of the tasks. In the first task, the emphasis is on identifying attributes of the pressure ulcers and on any documentation of patient noncompliance with steps to reduce pressure ulcers, such as pressure release. In the second task, the primary targets were the patients’ living situation, whether there was any evidence of malnutrition, and whether there was evidence of ongoing moisture on the skin. Annotation for each of the targeted variables included both a *cue* (phrase indicating presence) for the variable and the actual value assigned to the variable. The third annotation task was slightly different in that it emphasized labeling of the structural component of semistructured text (stored in templates). This information will allow for targeted extraction of the information contained in the template. Operational definitions of ICD-9-CM codes are being created. Once all of the various data are defined and extracted, they will be combined into an analytic dataset for development of risk models.

## Discussion

When completed, the results of this study will provide clinicians in the VHA system with a PrU risk assessment model specific to veterans with SCI. As part of the study, the predictive ability of the new model will be compared directly with that of the Braden Scale, the current risk assessment. Also, individual items on the Braden Scale will be tested for inclusion in the model. This will provide clinicians with information about the clinical utility of the new model. Initially, the new model will be distributed in the form of a simple stand-alone desktop computer program; however, our long-term goal is to deploy the risk assessment as an automated, integrated part of the VHA EHR. Clinicians could use the improved risk model to (1) maximize the impact of expensive resources for prevention of PrU (eg, specialty mattress and paid caregiver) by identifying those veterans at highest risk, (2) justify allocation of staffing resources (eg, home health care or telehealth), and (3) institute policies (eg, frequency of turning the veteran in bed). Further, we believe this study represents a model of how to leverage information from the EHR for risk assessment that could be applied to other clinical problems.

## Abbreviations

AHRQ: Agency for Healthcare Research and Quality
AORTIS: Aggregation, Organization, Reduction, Transformation, Interpretation, and Synthesis
ASIA: American Spinal Cord Injury Association
AUC: area under the curve
AUDIT-C: Alcohol Use Disorders Identification Test—Consumption
EHR: electronic health record
FY: fiscal year
GAM: generalized additive model
GATE: General Architecture for Text Engineering
HCPCS: Healthcare Common Procedural Coding System
HCUP: Healthcare Cost and Utilization Project
hx: history
ICD-9-CM: International Classification of Diseases, Ninth Revision, Clinical Modification
ICD-10-CM: International Classification of Diseases, Tenth Revision, Clinical Modification
IE: information extraction
info: information
LOINC: Logical Identifiers Names and Codes
LS: living situation
MN: malnutrition
MO: moisture
NLP: natural language processing
pAUC: partial area under the curve
PrU: pressure ulcer
PrU+: presence of a pressure ulcer
PrU-: absence of a pressure ulcer
ROC: receiver operating characteristic
SCI: spinal cord injury
SCI/D: Spinal Cord Injury and Disorder
VHA: Veterans Health Administration
VINCI: Veterans Affairs Informatics and Computing Infrastructure
VSSC: VHA Support Service Center
